# Global miRNA expression profiling of domestic cat livers following acute *Toxoplasma gondii* infection

**DOI:** 10.18632/oncotarget.16108

**Published:** 2017-03-10

**Authors:** Wei Cong, Xiao-Xuan Zhang, Jun-Jun He, Fa-Cai Li, Hany M. Elsheikha, Xing-Quan Zhu

**Affiliations:** ^1^ State Key Laboratory of Veterinary Etiological Biology, Lanzhou Veterinary Research Institute, Chinese Academy of Agricultural Sciences, Lanzhou, Gansu Province 730046, PR China; ^2^ College of Marine Science, Shandong University at Weihai, Weihai, Shandong Province 264209, PR China; ^3^ Faculty of Medicine and Health Sciences, School of Veterinary Medicine and Science, University of Nottingham, Sutton Bonington Campus, Loughborough, LE12 5RD, UK

**Keywords:** Toxoplasma gondii, domestic cat, liver, mircoRNA, RNA-seq

## Abstract

Although microRNAs (miRNAs) play an important role in liver homeostasis, the extent to which they can be altered by *Toxoplasma gondii* infection is unknown. Here, we utilized small RNA sequencing and bioinformatic analyses to characterize miRNA expression profiles in the liver of domestic cats at 7 days after oral infection with *T. gondii* (Type II) strain. A total of 384 miRNAs were identified and 82 were differentially expressed, of which 33 were up-regulated and 49 down-regulated. Also, 5690 predicted host gene targets for the differentially expressed miRNAs were identified using the bioinformatic algorithm miRanda. Gene ontology analysis revealed that the predicted gene targets of the dysregulated miRNAs were significantly enriched in apoptosis. Kyoto Encyclopedia of Genes and Genomes analysis showed that the predicted gene targets were involved in several pathways, including acute myeloid leukemia, central carbon metabolism in cancer, choline metabolism in cancer, estrogen signaling pathway, fatty acid degradation, lysosome, nucleotide excision repair, progesterone-mediated oocyte maturation, and VEGF signaling pathway. The expression level of 6 upregulated miRNAs (mmu-miR-21a-5p, mmu-miR-20a-5p, mmu-miR-17-5p, mmu-miR-30e-3p, mmu-miR-142a-3p, and mmu-miR-106b-3p) was confirmed by stem-loop quantitative reverse transcription PCR, which yielded results consistent with the sequencing data. These findings expand our understanding of the regulatory mechanisms of miRNAs underlying *T. gondii* pathogenesis and contribute new database information on cat miRNAs, opening a new perspective on the prevention and treatment of *T. gondii* infection.

## INTRODUCTION

*Toxoplasma gondii* is a highly prevalent apicomplexan protozoan parasite, which can cause serious clinical illnesses in humans and animals [1]. It has been reported to chronically infect roughly one-third of the world's human population [2]. *T. gondii* acquired during pregnancy may cause damage to the fetus and reactivation of latent infection can cause life-threatening encephalitis in immune-compromised individuals [3]. This parasite has an indirect two-host lifecycle, which is composed of asexual reproductive phase in the intermediate host and sexual reproductive phase in the definitive host (members of the Felidae family). The enteroepithelial sexual cycle of *T. gondii* is completed within 3 to 10 days after ingestion of intermediate host tissue containing *T. gondii* cysts. *T. gondii* can also spread throughout the cat's body and affect many organs [4–7]. Hence, cats are unique in respect of their ability to accommodate both sexual and asexual reproductions of *T. gondii*, making cats a significant source of infection to humans and animals [2, 8].

Besides the adverse clinical consequences on humans and other intermediate hosts *T. gondii* can cause disseminated and fatal infection in cats [4–7, 9]. Although any organ in the cat's body can be affected, clinical cases related to hepatic and pulmonary damage are particularly important because they are associated with quicker mortality [6, 10–12]. Also, liver dysfunction, enlargement, icterus, cholangiohepatitis, vomiting, abdominal effusion, and ascites are complications that frequently occur in *T. gondii*-infected cats. Therefore, with the great need for the development of efficacious treatment interventions (due to the lack of a vaccine and limited efficacies of current therapeutics), it is important to identify the molecular mechanisms that underpin liver damage caused by *T. gondii* infection. However, information about the molecular pathways that regulate the interaction between *T. gondii* and hepatic tissues has been limited to a few studies [13, 14].

The feline genome already encodes roughly 3,182 microRNA (miRNA) homologues, which can regulate the expression of signalling cascades that perform key cellular functions, such as cell cycle regulation, proliferation, differentiation, apoptosis, and carcinogenesis. miRNAs constitute a group of endogenous non-coding small RNAs (18 to 25 nucleotides [nt] long) that regulate gene expression by binding to mRNA and inhibiting translation [15–18]. miRNAs play an important role in liver homeostasis, and aberrant expression of miRNAs has been associated with a variety of liver diseases, such as viral hepatitis, hepatocellular carcinoma and fatty liver disease [19]. Alterations of host miRNA expression have also been observed in some parasitic infections, such as *Cryptosporidium parvum*, *Plasmodium falciparum* and *T. gondii* (reviewed in [20]), underscoring the potential role of miRNAs in mediating the interaction between *T. gondii* and host cells. Despite the impact of *T. gondii* infection on hepatic function the mechanisms underlying the alterations of hepatic miRNAs expression following acute *T. gondii* infection remain poorly understood.

In this study, we hypothesized that *T. gondii* infection alters the expression of hepatic miRNAs and that differentially expressed miRNAs mediate the interaction between *T. gondii* and cat's liver. Here we use genome-wide, small RNA sequencing to characterize the global miRNA transcriptional response of feline liver to infection with *T. gondii* (Type II) PRU strain. Our study provides a full picture of the hepatic miRNA repertoire during acute *T. gondii* infection in domestic cats, including novel miRNAs, involved in host cell cycle, apoptosis and anti-*T. gondii* defense.

## RESULTS

### Confirmation of *T. gondii* infection in the cat livers

Under the conditions we used positive PCR results were obtained, providing laboratory confirmation of *T. gondii* infection in the livers of infected cats. RFLP analysis of the positive PCR amplicons of *T. gondii B1* gene revealed a restriction fragment pattern characteristic to *T. gondii* genotype II. The livers of control cats and negative PCR control yielded negative PCR results.

### Analysis of miRNA expression

**Figure 1:**
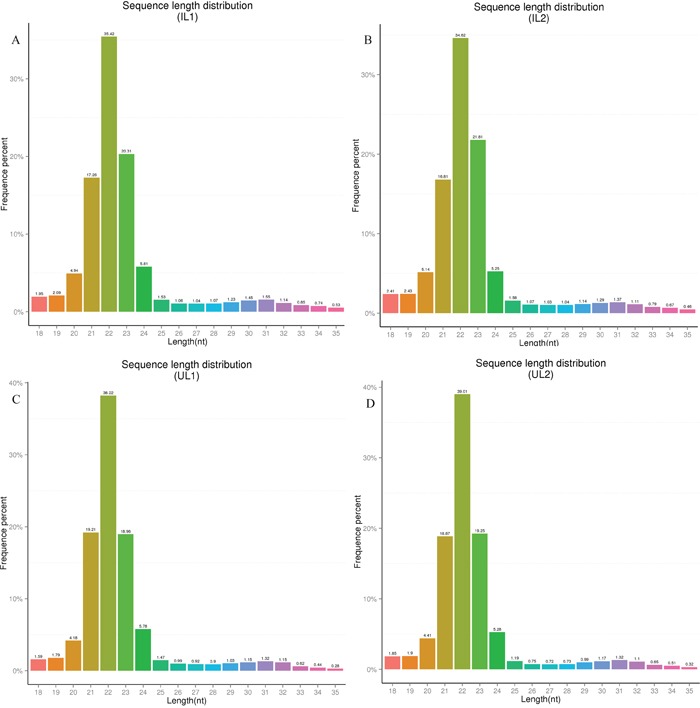
Length distribution of the small RNA expressions in the liver of domestic cats infected with *Toxoplasma gondii* compared to control cats **(A** and **B)** Group 1 and group 2 of *T. gondii*-infected domestic cats; **(C** and **D)** Group 1 and group 2 of uninfected, control, domestic cats. Abbreviations: IL1, IL2, UL1, and UL2: infected liver 1, infected liver 2, uninfected liver 1, uninfected liver 2, respectively.

**Figure 2:**
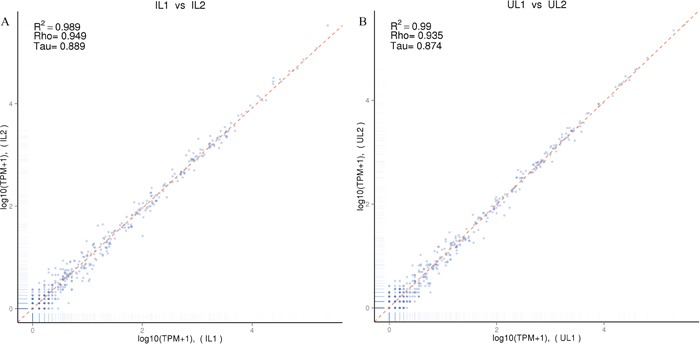
Correlation analysis of the small RNA expressions in the livers of *T. gondii*-infected and uninfected cats **(A)** Correlation analysis of small RNA expression levels between *T. gondii*-infected cats of group 1 and group 2 (IL1 vs IL2); **(B)** Correlation analysis of small RNA expression levels between uninfected cats of group 1 and group 2 (Ul1 vs UL2).

miRNA libraries of livers from two *T. gondii*-infected or two control cat groups were successfully sequenced and sequencing data is summarized in Table 1 and Table 2. Length distributions of clean reads in the libraries were between 20-24 nt (Figure 1A-1D). A very high intra-group correlation was detected between the two miRNA libraries of *T. gondii*-infected liver samples (R^2^ =0.989) (Figure 2A) and the two miRNA libraries of uninfected liver samples (R^2^ =0.99) (Figure 2B). The known and novel mature miRNAs in *T. gondii*-infected and control groups were summarized in Table 3 and Table 4. Finally, through comparing *T. gondii*-infected and uninfected sRNA libraries, 82 differentially expressed miRNAs were identified, including 33 up-regulated and 49 down-regulated miRNAs (Table 5).

**Table 1:** Summary of small RNA sequencing data obtained in the present study

**Library type**	**Reads**	**Bases**	**Error rate**	**Q20**	**Q30**	**GC content**
Infected liver Group 1	10665548	0.533G	0.01%	96.21%	92.13%	49.65%
Infected liver Group 2	12569494	0.628G	0.01%	96.16%	92.13%	49.98%
Uninfected liver Group 1	11727706	0.586G	0.01%	96.24%	92.33%	49.56%
Uninfected liver Group 2	10273236	0.514G	0.01%	96.44%	92.60%	49.72%

**Table 2:** Summary of the standard bioinformatic quality check and cleaning of small RNAs

**Library**	**Infected groups**		**Uninfected groups**	
	**Infected liver Group 1**	**Infected liver Group 2**	**Uninfected liver Group 1**	**Uninfected liver Group 2**
Total reads	10665548 (100.00%)	12569494 (100.00%)	11727706 (100.00%)	10273236 (100.00%)
N%>10%	531 (0.00%)	636 (0.01%)	606 (0.01%)	540 (0.01%)
Low quality	40551 (0.38%)	47641 (0.38%)	42076 (0.36%)	32984 (0.32%)
5_adapter_contamine	1247 (0.01%)	1565 (0.01%)	1026 (0.01%)	915 (0.01%)
3_adapter_null or insert_null	174157 (1.63%)	176650 (1.41%)	223708 (1.91%)	195007 (1.90%)
With ployA/T/G/C	10772 (0.10%)	12621 (0.10%)	7991 (0.07%)	6939 (0.07%)
Clean reads	10438290 (97.87%)	12330381 (98.10%)	11452299 (97.65%)	10036851 (97.70%)

**Table 3:** Known miRNA mapping

**Criteria**	**Infected groups**		**Uninfected groups**	
	**Infected liver Group 1**	**Infected liver Group 2**	**Uninfected liver Group 1**	**Uninfected liver Group 2**
Mapped mature	358	360	348	356
Mapped hairpin	273	274	266	268
Mapped uniq sRNA	2531	2604	2411	2409
Mapped total sRNA	3086174	3665542	3484600	3037510

**Table 4:** Novel miRNA mapping

**Types**	**Infected liver Group 1**	**Infected liver Group 2**	**Uninfected liver Group 1**	**Uninfected liver Group 2**
Novel mature	69	76	75	73
Novel star	10	14	12	10
Novel hairpin	72	78	80	77
Mapped uniq sRNA	186	187	161	161
Mapped total sRNA	12381	12103	13702	7981

**Table 5:** Differentially expressed miRNAs

**Type**	**miRNA**	**Fold change**	***P*-value**	***P*-adjustment**
Up-regulated	mmu-miR-21a-5p	2.564	1.32E-17	3.15E-15
	mmu-miR-20a-5p	1.610	3.26E-09	3.88E-07
	mmu-miR-339-5p	1.839	5.42E-06	0.0002
	mmu-miR-101a-3p	1.855	8.22E-06	0.0002
	mmu-miR-320-3p	1.507	9.27E-06	0.0002
	mmu-miR-195a-5p	1.805	1.33E-05	0.0003
	mmu-miR-126a-3p	1.486	2.05E-05	0.0003
	mmu-miR-23a-3p	1.585	2.13E-05	0.0003
	mmu-miR-140-3p	1.701	2.45E-05	0.0003
	mmu-miR-28a-5p	1.500	0.0002	0.0018
	mmu-miR-223-3p	1.917	0.0002	0.0023
	mmu-miR-30f	1.504	0.0002	0.0023
	mmu-miR-126a-5p	1.437	0.0003	0.0025
	mmu-miR-126b-3p	1.435	0.0003	0.0025
	mmu-miR-17-5p	1.436	0.0005	0.0036
	mmu-miR-19b-3p	1.485	0.0007	0.0048
	mmu-miR-27a-5p	1.741	0.0011	0.0076
	mmu-miR-486b-3p	1.593	0.0011	0.0076
	mmu-miR-151-3p	1.338	0.0017	0.0093
	mmu-miR-27a-3p	1.389	0.0017	0.0093
	mmu-miR-486a-3p	1.571	0.0017	0.0093
	mmu-miR-99b-5p	1.359	0.0021	0.0106
	novel_1	1.998	0.0030	0.0134
	mmu-miR-3074-5p	1.315	0.0049	0.0202
	mmu-miR-30e-3p	1.325	0.0052	0.0211
	mmu-miR-24-3p	1.306	0.0055	0.0212
	mmu-miR-101c	1.052	0.0069	0.0252
	mmu-miR-378c	1.528	0.0069	0.0252
	mmu-miR-486a-5p	1.494	0.0075	0.0271
	mmu-miR-361-3p	1.379	0.0101	0.0342
	mmu-miR-106b-3p	1.255	0.0153	0.0451
	mmu-miR-142a-3p	1.690	0.0152	0.0451
	mmu-miR-142b	1.690	0.0152	0.0451
Down-regulated	mmu-let-7f-5p	-1.546	1.73E-07	1.38E-05
	mmu-let-7i-5p	-1.705	1.37E-06	6.52E-05
	mmu-miR-365-3p	-1.532	1.24E-06	6.52E-05
	mmu-miR-148a-3p	-1.662	6.17E-06	0.0002
	mmu-miR-381-3p	-2.264	4.87E-06	0.0002
	mmu-miR-370-3p	-2.876	8.54E-06	0.0002
	mmu-miR-3071-5p	-2.007	1.62E-05	0.0003
	mmu-miR-136-3p	-1.987	2.23E-05	0.0003
	mmu-miR-30c-2-3p	-1.683	6.94E-05	0.0009
	mmu-miR-128-3p	-1.487	7.45E-05	0.0009
	mmu-miR-30d-5p	-1.358	0.0002	0.0018
	mmu-let-7a-5p	-1.426	0.0002	0.0023
	mmu-miR-340-5p	-1.570	0.0002	0.0023
	mmu-let-7g-5p	-1.349	0.0003	0.0023
	mmu-miR-493-5p	-2.565	0.0003	0.0023
	mmu-miR-218-5p	-1.618	0.0003	0.0025
	mmu-miR-127-3p	-1.517	0.0003	0.0026
	mmu-let-7e-5p	-1.380	0.0006	0.0042
	mmu-miR-98-5p	-1.452	0.0012	0.0077
	mmu-miR-409-3p	-1.919	0.0013	0.0083
	mmu-let-7c-5p	-1.572	0.0015	0.0089
	mmu-miR-139-5p	-1.418	0.0015	0.0089
	mmu-miR-129b-3p	-1.526	0.0017	0.0093
	mmu-miR-129-5p	-1.526	0.0018	0.0095
	mmu-miR-382-3p	-2.192	0.0019	0.0096
	mmu-miR-193b-3p	-1.554	0.0020	0.0102
	mmu-miR-429-3p	-1.738	0.0021	0.0102
	mmu-miR-181b-5p	-1.485	0.0023	0.0109
	mmu-miR-1b-5p	-1.752	0.0024	0.0110
	mmu-miR-1a-3p	-1.751	0.0024	0.0111
	mmu-miR-450a-5p	-1.536	0.0025	0.0112
	mmu-miR-423-3p	-1.296	0.0034	0.0148
	mmu-miR-328-3p	-1.452	0.0035	0.0148
	mmu-miR-532-5p	-1.316	0.0048	0.0200
	mmu-miR-148a-5p	-1.521	0.0055	0.0212
	mmu-miR-499-5p	-1.473	0.0055	0.0212
	novel_115	-2.137	0.0059	0.0223
	mmu-miR-425-5p	-1.490	0.0080	0.0283
	mmu-miR-299a-3p	-1.481	0.0091	0.0319
	mmu-miR-200a-3p	-1.500	0.0099	0.0342
	mmu-miR-30b-5p	-1.305	0.0104	0.0350
	mmu-miR-30e-5p	-1.486	0.0108	0.0356
	mmu-miR-200a-5p	-1.410	0.0110	0.0358
	mmu-miR-26a-5p	-1.260	0.0113	0.0363
	mmu-miR-99a-5p	-1.379	0.0125	0.0396
	mmu-miR-409-5p	-1.705	0.0127	0.0398
	mmu-miR-92a-3p	-1.350	0.0132	0.0409
	mmu-miR-129-1-3p	-1.769	0.0146	0.0446
	mmu-miR-148b-3p	-1.277	0.0167	0.0484

### Pathway analysis of miRNA targets

**Figure 3:**
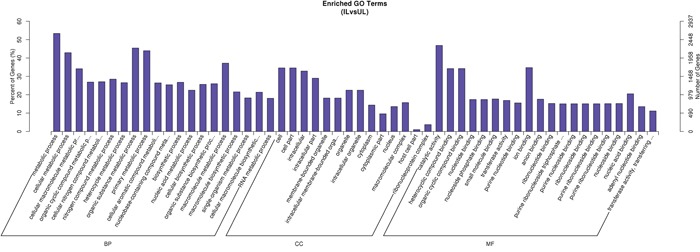
GO enrichment analysis of predicted gene targets of differentially expressed miRNAs Abbreviations: IL vs UL: infected liver vs uninfected liver, BP: biological process, MF: molecular function, CC: cellular component.

**Figure 4:**
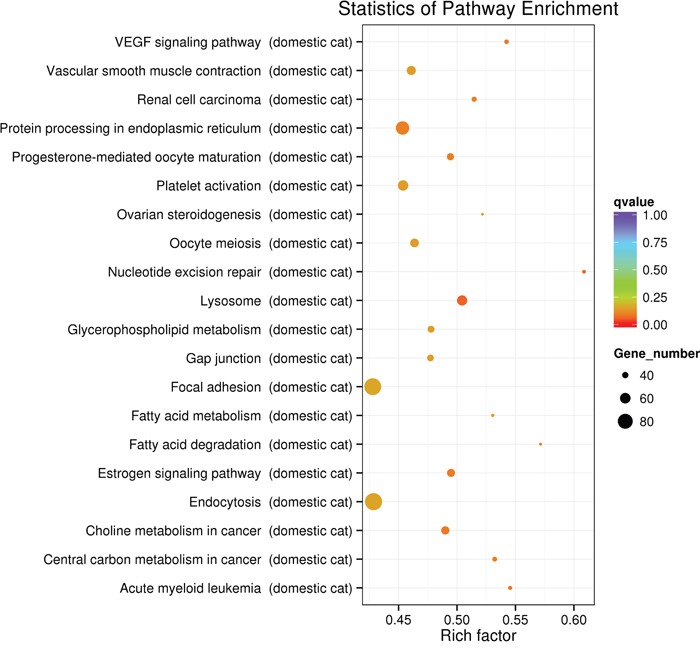
The top 20 enriched pathways of the predicted host targets of the differentially expressed miRNAs

A total of 5690 predicted host targets were identified (Supplementary Table 2). Based on the predicted targets of the differentially expressed miRNAs GO enrichment analysis was performed in order to identify the biological processes, molecular functions and cellular components. The enriched GO terms of the biological processes, molecular functions and cellular components are shown in Figure 3, respectively. KEGG enrichment analysis showed that target genes were related to multiple pathways, including nucleotide excision repair, lysosome, vascular endothelial growth factor (VEGF) signaling, estrogen signaling, acute myeloid leukemia, central carbon metabolism in cancer, choline metabolism in cancer, fatty acid degradation, progesterone-mediated oocyte maturation, and renal cell carcinoma. The top 20 KEGG enrichment pathways are shown in Figure 4.

### miRNA expression validation by qRT-PCR

Six miRNAs, including mmu-miR-21a-5p, mmu-miR-20a-5p, mmu-miR-17-5p, mmu-miR-30e-3p, mmu-miR-142a-3p, and mmu-miR-106b-3p, were selected for confirmation by real time PCR to verify the expression levels of the differentially expressed miRNAs using miRNA specific primers (Supplementary Table 1). The results confirmed that these selected miRNAs were differentially expressed between infected livers and uninfected livers and were consistent with the results obtained by RNA-sequencing analysis (Supplementary Figure 1). Data set from the RNA-sequencing experiment has been deposited in the GEO database under accession number PRJNA356106.

## DISCUSSION

Previous studies showed that *T. gondii* infection can alter the expression of host miRNAs, indicating that miRNAs may be involved in the pathogenesis of *T. gondii* infection [21–24]. Our results have also shown that acute *T. gondii* infection alters the level of miRNAs in the liver of domestic cats. In the present study, we used small RNA sequencing to identify cellular miRNAs and signaling pathways involved in the response of cats to *T. gondii* infection. Specifically, we determined miRNAs that are differentially expressed by comparing sham-infected to *T. gondii*-infected cat livers at 7 days after infection. This analysis identified 82 differentially expressed microRNAs, of which 33 were increased and 49 decreased. By using qRT-PCR, the expression level of six up-regulated miRNAs (mmu-miR-21a-5p, mmu-miR-20a-5p, mmu-miR-17-5p, mmu-miR-30e-3p, mmu-miR-142a-3p, and mmu-miR-106b-3p) were consistent with the results obtained by sequencing analysis. Knowledge of molecular changes in human liver during the acute phase of toxoplasmosis is lacking due to the mild and subtle nature of this infection especially in immune-component individuals [1, 2]. Hence, these findings may serve as the basis for understanding the molecular mechanisms associated with hepatic pathology during acute *T. gondii* infection.

Differentially expressed miRNAs were associated with signalling pathways involved mainly in cell cycling, apoptosis, oncogenesis, and host defense. Among the differentially expressed miRNAs, miR-21a-5p, miR-17-5p, miR-223-3p, miR-27a-5p, miR-126, and miR-486 were significantly upregulated in *T. gondii*-infected livers compared to controls. Of note, the level of miR-21a-5p expression was elevated in various cancer tissues, including rectal, gastric and lung tissues [25–27] and has been suggested to play a role in tumor biology [27]. The similarity between the expression of miR-21a-5p during *T. gondii* infection and various forms of cancers is interesting. One striking finding was the correlative link between upregulation of miR-17-92 in *T. gondii*-infected human foreskin fibroblasts [28] and in human astrocyticglioma tissue [29]. The presence of RNA silencing machinery and small silencing RNAs in *T. gondii* genome [30] indicates that this parasite has the ability to use its own miRNAs to interrupt host cell functions in analogy to oncogenic viruses [31].

miR-17-5p, a key regulator of the G1/S phase cell cycle transition, was up-regulated in our study in agreement with others who reported overexpression of miR-17-5p in human and mouse spleen in response to *T. gondii* infection [21, 31]. *T. gondii* can increase miR-17∼92 and miR-106b∼25 that play key roles in the regulation of mammalian cell cycle by influencing the functionally intertwined pathways of apoptosis and G1/S cell cycle progression [32]. miR-17-5p targets mouse Bcl2l11, Zmat3, Aifm1, and Capn2 to increase host apoptotic process and targets mouse Ppp3r1 and Akt3 to promote cellular apoptosis process [21]. Also, miR-17-5p may function as both a tumor suppressor [33] and as an oncogenic activator [34] by targeting both anti- and pro-proliferative genes and by competing with each other in different cellular contexts [35]. The effect of *T. gondii* infection on the expression of miRNAs (miR-30c-1, miR-125b-2, miR-23b-27b-24-1, and miR-17∼92 cluster genes) that have anti-apoptotic activity has been reported [36]. Modulating these apoptosis-related miRNAs with mimics or inhibitors can validate their roles in the dysregulation of host cell apoptotic machinery during *T. gondii* infection.

The miR-223-3p has been implicated in the regulation of inflammatory response [37] and granulocyte production and function [38], and can function as a tumor suppresser in osteosarcoma by regulating the osteosarcoma cell cycle progression and proliferation [39]. The level of miR-223-3p was significantly increased in infected samples, suggesting that *T. gondii* infection of feline liver stimulates the production of miR-223-3p, which plays a role in the activation of inflammatory response elicited in response to *T. gondii* infection. This is concordant with a previous study showing that miRNAs, such as miR-146a and miR-155, known to activate immune and inflammatory responses can influence host response to *T. gondii* infection [40–43]. Also, the upregulation of miR-27a-5p (a regulator of lipid metabolism-related genes) and miR-21-5p in the infected liver samples suggests that both miR-27a-5p and miR-21-5p play a role in host response to infection. This assumption is supported by the association between inhibition of miR-21 and increased *Cryptosporidium parvum* burden [29].

miR-126 is associated with tumorigenesis and has recently been found to modulate the survival and function of Plasmacytoid dendritic cells (pDCs) via positive regulation of the vascular endothelial growth factor (VEGF) signaling pathway [44]. miR-126 upregulation may activate Toll-like receptor (TLR)/MyD88 signalling in pDCs to secrete large amounts of type I interferons (IFNs), which is essential for host resistance to *T. gondii* infection. Also, the biological significance of *T. gondii*-induced upregulation of miR-486 may lie in its ability to augment the host defense mechanisms. miR-486 has been shown to activate nuclear factor (NF)-κB signaling pathway [45], which leads to the production of proinflammatory cytokines, thereby providing a protection against *T. gondii* infection. Of note, both miR-486 and the NF-κB signaling pathway have oncogenic roles in human cancers, such as glioma progression [45].

The let-7 family is a key regulator of the innate immune response. The level of let-7i during protozoal infection with *C. parvum* infection was found reduced together with increase of TLR4 in biliary epithelial cells, contributing to cholangiocyte's defense responses [46]. In line with this study our results revealed significant downregulation of mmu-let-7f-5p, mmu-let-7i-5p, mmu-let-7a-5p, mmu-let-7g-5p, mmu-let-7e-5p, and mmu-let-7c-5p in *T. gondii*-infected cat's livers compared with controls. Finally, the fact that miRNAs are host-, tissue-, and strain-specific [47] explains why the expression of some miRNA (e.g. miR-712-3p, miR-511-5p and miR-217-5p) that are dysregulated during *T. gondii* infection [48] was not altered in our study.

In conclusion, these findings provided new insight regarding the ability of *T. gondii* to alter the expression of 82 microRNAs in cat liver. Our study revealed that through reprogramming of hepatic miRNAs expression *T. gondii* influences the cellular microenvironment and host anti-*T. gondii* response, which are likely to play roles in the parasite pathogenesis. GO analysis revealed that the predicted targets of the differentially expressed miRNAs were involved in the regulation of cell cycle and most of the identified KEGG pathways were related to cancer. Considering the immunoregulatory effects of miRNAs and their ability to modulate crucial host cellular targets needed for *T. gondii* replication, miRNAs may hold promise as biomarkers for prediction of disease progression. Finally, miRNA pathways that are stimulated during infection may offer potential targets for therapeutic control of toxoplasmosis.

## MATERIALS AND METHODS

### Ethics statement

This study was performed in strict accordance with the recommendations set forth in the Animal Ethics Procedures and Guidelines of the People's Republic of China. All animal experiments were reviewed and approved by the Animal Ethics Committee of Lanzhou Veterinary Research Institute, Chinese Academy of Agricultural Sciences (Approval No. LVRIAEC2014-009). Liver tissue collection was performed as a terminal procedure under isoflurane anesthesia and all efforts were made to minimize suffering.

### Animals, parasite infection and sample collection

Twelve, 3 month-old, domestic cats (*Felis catus*) of the Chinese Li Hua breed were purchased from a local breeder and were housed in a controlled environment. The cats belonged to two litters, six cats per litter. These 12 cats were randomly allocated to four groups (two infected and two control) with three cats per group. Before the experiment, all cats were confirmed to be free from *T. gondii* using the modified agglutination test and free of major viral infections (e.g. feline calicivirus and coronavirus, feline immunodeficiency virus, feline leukemia virus, and feline parvovirus) based on serological examination. Cats were maintained on commercial cat diets (Royal Canine Inc., St. Charles, MO, USA) and water *ad libitum* during the 3 weeks prior to experimentation in order to allow cats to acclimate and to minimize any potential dietary influence on the study results. During the experiment cats were individually fed once daily based on daily energy requirements and water was available *ad libitum*.

*Toxoplasma gondii* strain used in this study was the PRU strain (Genotype II), which is maintained in our laboratory by passage through Kunming mice as described previously [49]. *T. gondii* type II was used in this study because it seems to be the predominant genotype circulating in cats [50–52]. Also, the PRU strain is able to produce brain tissue cysts in mouse and oocysts in the gut of cats and is thus a suitable candidate for a standardized challenge model in cats. The number of *T. gondii* cysts was determined using an optical microscope and was adjusted to 100 cysts mL^−1^ in phosphate buffered saline (PBS), pH 7.4. Each cat was infected by intragrastric inoculation with 100 cysts in 1 mL PBS. Control cats were sham-infected with PBS only. Livers were harvested 7 days post infection (7 dpi) in order to allow sufficient time for the infection to be established in the liver [1]. Collected livers were rinsed extensively in saline, flash frozen in liquid nitrogen, and stored at −80°C until processing.

### Detection of infection in the liver

Genomic DNA was extracted from the collected liver samples using TIANamp Genomic DNA kit according to the manufacturer's recommendations (TianGen™, Beijing, China). Then, a semi-nested PCR targeting *T. gondii B1* gene was performed to detect *T. gondii* infection [53]. DNA samples giving positive *B1* amplification were genotyped using PCR-restriction fragment length polymorphism analysis as described previously [54].

### RNA extraction and qualification

Total RNA was prepared individually from the cryo-preserved liver tissues of the cats using TRIzol Reagent according to the manufacturer's instructions (Invitrogen Co. Ltd). RNA degradation and contamination was checked on 1% agarose gels. RNA purity was evaluated using the NanoPhotometer® spectrophotometer (IMPLEN, CA, USA). RNA concentration was determined using Qubit® RNA Assay Kit in Qubit® 2.0 Flurometer (Life Technologies, CA, USA). RNA integrity was assessed using the RNA Nano 6000 Assay Kit of the Agilent Bioanalyzer 2100 system (Agilent Technologies, CA, USA).

### RNA sequencing library preparation and transcriptomic analysis

RNA samples from *T. gondii*-infected and non-infected livers collected at 7 dpi were sent to Beijing Novogene Bioinformatics Institute for Illumina sequencing. To analyze miRNAs by sequencing, a total of 3 μg RNAs of three pooled samples from each group were used for the construction of four small RNA (sRNA) libraries, which were subjected to sequencing on a Hi-seq 2500 platform. Raw data (raw reads) of fastq format were firstly processed through custom perl and python scripts. In this step, clean reads were obtained by removing reads containing ploy-N, with 5′ adapter contaminants, without 3′ adapter or the insert tag, containing ploy A or T, or G or C and low quality reads from raw data. At the same time, Q20, Q30 and GC-content of the raw data were calculated. Then, we chose a certain range of length from clean reads to do all downstream analyses. Next, the small RNA tags were mapped to the feline reference genome sequence using Bowtie software [55]. The following parameters were used: -k [valid alignments per read], 1; -m [number of possible alignments], 10; -l [seed length], 25; --best [optimal alignments]).

Mapped small RNA tags were used to look for known miRNA. miRBase20.0 was used as reference, and modified software mirdeep2 [56] and srna-tools-cli were used to obtain the potential miRNA and draw the secondary structures. Custom scripts were used to obtain the miRNA counts and base bias on the first position of identified miRNA with certain length and on each position of all identified miRNA, respectively. To remove tags originating from protein-coding genes, repeat sequences, rRNA, tRNA, snRNA, and snoRNA, small RNA tags were mapped to RepeatMasker, Rfam database or those types of data from the specified species itself.

The available software miREvo [57] and mirdeep2 [56] were integrated to predict novel miRNA through exploring the secondary structure, the dicer cleavage site and the minimum free energy of the small RNA tags unannotated in the former steps. miRNA expression levels were estimated with TPM (transcript per million) units [58]. Differential expression analysis of infected versus control groups was performed using the DESeq R package (1.8.3). The *P*-values was adjusted using the Benjamini & Hochberg method. Corrected *P*-value of 0.05 was set as the threshold for significantly differential expression by default.

Predicting the target gene of miRNA was performed by psRobot_tar in miRanda [59]. Gene Ontology (GO) enrichment analysis was used to categorize the target genes of the differentially expressed miRNAs. GOseq based Wallenius non-central hyper-geometric distribution [60], which can adjust for gene length bias, was implemented for GO enrichment analysis. The enrichment of target genes in KEGG pathways were tested by the software KOBAS [61].

### Validation of miRNA expression

The stem-loop quantitative reverse transcription PCR was used to validate the results of miRNA expression analysis as described previously [21, 62]. Stem-loop RT-PCR was performed on ABI PRISM® 7500 Sequence Detection System using SYBR Green qPCRSuperMix according to the manufacturer's protocol (Invitrogen). All qRT-PCR reactions were performed in three replicates. Gene expressions were calculated by 2^−ΔΔCT^ relative expression method as previously described [63]. snRNA U6 was used as normalization control in qRT-PCR.

## SUPPLEMENTARY MATERIALS FIGURE AND TABLES




