# Supplementary material for: Global miRNA expression profiling of domestic cat livers following acute Toxoplasma gondii infection
Source: Oncotarget. 2017 Mar 10;8(15):25599–611. doi: 10.18632/oncotarget.16108 (PMC5421954; doi:10.18632/oncotarget.16108)
Supplement: Supplementary file 1 [file oncotarget-08-25599-s001.pdf]

Global miRNA expression profiling of domestic cat livers following acute *Toxoplasma gondii* infection

SUPPLEMENTARY MATERIALS

SUPPLEMENTARY FIGURE AND TABLES

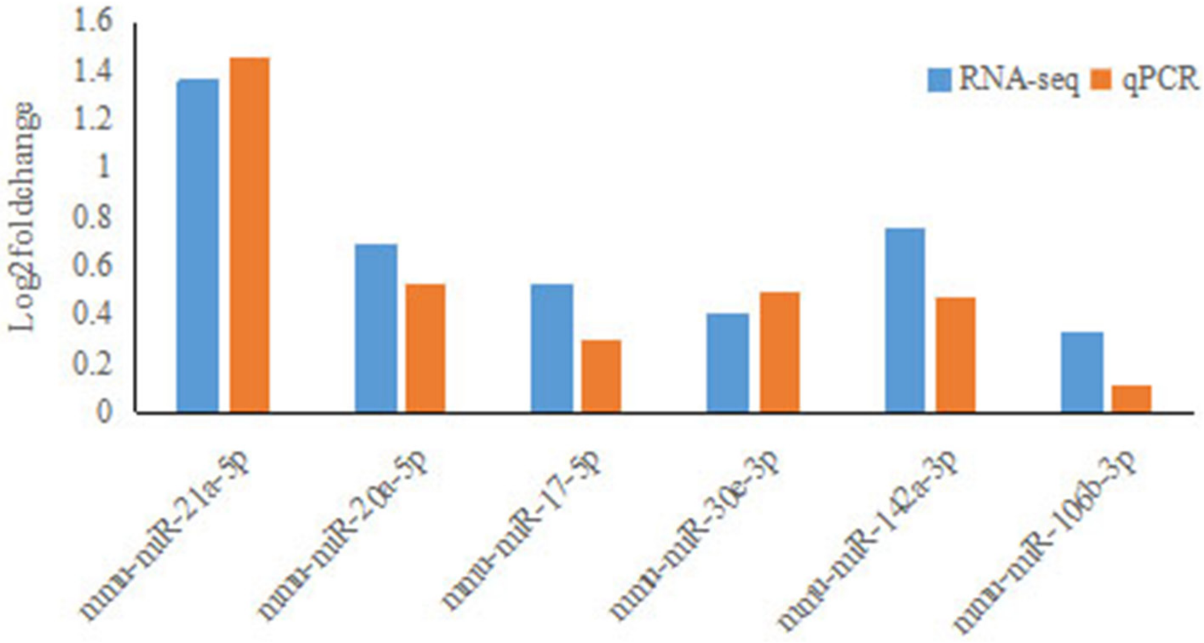

Supplementary Figure 1: Validation of the differential expression of 6 miRNAs identified in the sequencing analysis using stem-loop qRT-PCR. U6 was used as a reference gene.

Supplementary Table 1: Primers for stem-loop qRT-PCR

Primer name	Primer sequence (5′ to 3′)
mmu-miR-21a-5p F	ACACTCCAGCTGGGTAGCTTATCAGACTGATG
mmu-miR-20a-5p F	ACACTCCAGCTGGGTAAAGTGCTTATAGTGCAG
mmu-miR-17-5p F	ACACTCCAGCTGGGCAAAGTGCTTACAGTGCAG
mmu-miR-30e-3p F	ACACTCCAGCTGGGCTTTCAGTCGGATGTTTA
mmu-miR-142a-3p F	ACACTCCAGCTGGGTGTAGTGTTTCCTACTTTA
mmu-miR-106b-3p F	ACACTCCAGCTGGGCCGCACTGTGGGTACTTG
miRNA-R[a]	CTCAACTGGTGTCGTGGA
U6-F	CTCGCTTCGGCAGCACA
U6-R	AACGCTTCACGAATTTGCGT

[a] miRNA-R: used as universal primer for miRNA quantification.

Supplementary Table 2: Predicted gene targets of differentially expressed miRNAs

See Supplementary File 1
